# Dose-dependent association between elevated resting IL-6 levels and severity of mental stress-induced myocardial perfusion defects

**DOI:** 10.1017/S0033291726103638

**Published:** 2026-04-07

**Authors:** Han Yin, Jinna Chang, Mengyang Jia, Cheng Jiang, Yuanhao Wang, Fengyao Liu, Bingqing Bai, Yuting Liu, Quanjun Liu, Haochen Wang, Jingnan Huang, Dahui Xue, Nana Jin, Lixin Cheng, Jingjin Liu, Shuxia Wang, Huan Ma, Qingshan Geng

**Affiliations:** 1 Department of Cardiology, Shenzhen People’s Hospital (The Second Clinical Medical College, Jinan University, The First Affiliated Hospital, Southern University of Science and Technology), Shenzhen, China; 2 Guangdong Cardiovascular Institute, Guangdong Provincial People’s Hospital, Guangdong Academy of Medical Sciences, Guangzhou, China; 3 School of Medicine, Southern University of Science and Technology, Shenzhen, China; 4 Department of Geriatrics, Shenzhen People’s Hospital (The Second Clinical Medical College, Jinan University, The First Affiliated Hospital, Southern University of Science and Technology), Shenzhen, China; 5 Department of Nuclear Medicine, Guangdong Provincial People’s Hospital, Guangdong Academy of Medical Sciences, Southern Medical University, Guangzhou, China

**Keywords:** angina with no obstructive coronary artery disease, IL-6, mental stress-induced myocardialischemia, myocardial perfusion

## Abstract

**Background:**

Mental stress-induced myocardial ischemia (MSIMI) is common in women with angina with no obstructive coronary artery disease (ANOCA) and is associated with adverse cardiovascular outcomes. Interleukin-6 (IL-6), a central mediator of chronic inflammation, predicts future cardiovascular risk, but its relationship with MSIMI remains unclear.

**Methods:**

Eighty women with ANOCA underwent ^13^N-ammonia positron emission tomography/computed tomography to assess myocardial perfusion and myocardial blood flow (MBF) at rest, during mental stress, and during adenosine-induced stress. Resting inflammatory biomarkers were measured, and multivariable logistic and linear regression models were used to evaluate associations with mental stress-induced perfusion defects. Proteomic profiling was performed in a selected subset to explore potential underlying mechanisms.

**Results:**

Mental stress induced significantly greater myocardial perfusion defects in MSIMI+ patients. Resting IL-6 levels were significantly higher in MSIMI+ patients (3.20 versus 1.80 pg/mL, *p* = 0.024). Although baseline CRP, hsCRP, and complement C3 levels were also higher in MSIMI+ patients, only resting IL-6 remained independently associated with both the presence of MSIMI and the severity of mental stress-induced myocardial perfusion defects after adjustment for demographic, clinical, and psychosocial factors, and resting MBF. Proteomic analyses demonstrated enrichment of innate immune–hemostatic pathways and mitochondrial oxidative phosphorylation in MSIMI+ patients with high IL-6 levels.

**Conclusions:**

Elevated resting IL-6 is independently associated with the presence and severity of MSIMI in women with ANOCA. These findings suggest that IL-6 may serve as a biomarker of MSIMI and support IL-6-related inflammation as a key pathophysiological pathway underlying MSIMI, with potential implications for targeted therapeutic strategies.

## Introduction

Mental stress (MS) has emerged as an important determinant of prognosis in individuals with cardiovascular and cerebrovascular disease (Cohen, Janicki-Deverts, & Miller, 2007; Kivimäki & Steptoe, 2018). In patients with chronic stable coronary artery disease (CAD) or angina with no obstructive coronary artery disease (ANOCA), not only physical exertion but also MS can provoke myocardial ischemia, a phenomenon termed mental stress-induced myocardial ischemia (MSIMI) (Jin, Cheng, & Geng, 2024; Ma et al., 2023; Rozanski et al., 1988). MSIMI has been associated with a more than twofold increased risk of future adverse cardiovascular events, yet its underlying mechanisms remain incompletely understood (Vaccarino et al., 2021). Previous studies have noted several correlates of MSIMI – such as a higher prevalence of depression, anxiety, and post-traumatic stress; greater peripheral vasoreactivity; and exaggerated hemodynamic responses – but the deeper biological pathways remain insufficiently characterized (Martin et al., 2023; Sherin & Nemeroff, 2011).

Inflammatory activation is a well-recognized mechanism linking psychosocial stress and mental health disorders to cardiovascular dysfunction (Hinterdobler, Schunkert, Kessler, & Sager, 2021; Li et al., 2025). On the one hand, MS and negative emotional states have been shown to elevate circulating interleukin-6 (IL-6) – a pleiotropic cytokine central to the inflammatory cascade – as well as other pro-inflammatory mediators (Dentino et al., 1999; Kiecolt-Glaser et al., 2003). On the other hand, chronic, low-grade sterile inflammation is a key driver of adverse cardiovascular outcomes, potentially through impaired endothelial function, accelerated atherogenesis, and cytokine-mediated increases in vascular smooth muscle contractility (Furman et al., 2019; G. Liu et al., 2025; Peng, Shu, Zhang, & Wang, 2019). Heightened inflammatory activity further enhances leukocyte and platelet reactivity, fostering microvascular obstruction and coronary vasospasm, thereby increasing susceptibility to ischemia (Stokes & Granger, 2012). For example, IL-6 itself plays a central role in promoting endothelial dysfunction, atherosclerotic progression, and downstream cardiovascular events (Batra et al., 2021; Held et al., 2017; Wassmann et al., 2004). Prior studies have also shown that individuals with elevated baseline inflammatory burden – such as higher C-reactive protein (CRP) levels – are more likely to develop stress-provoked ischemia (Shah et al., 2006), raising the possibility that resting inflammatory status may be a fundamental determinant of MSIMI susceptibility.

Leveraging data from the Mental Stress in Women Study (Ma et al., 2023), which assessed resting inflammatory markers (IL-6, CRP, and a panel of immune- and inflammation-related markers) in an ANOCA population and quantified myocardial perfusion responses to MS using ^13^N-ammonia positron emission tomography/computed tomography (PET/CT), we sought to clarify whether an association exists between baseline inflammatory status and MS-induced perfusion abnormalities. In parallel, through proteomic analyses of plasma samples, we aimed to explore potential biological processes underlying MSIMI, providing new insights into its pathogenesis and potential therapeutic targets.

## Methods

### Study design

The detailed methodology and the main results of the trial have been previously published (Ma et al., 2020, 2023). Briefly, 84 women with ANOCA and 42 age-matched healthy controls were enrolled in the Mental Stress in Women Study, conducted at the Guangdong Provincial People’s Hospital between June 2019 and April 2021. The primary objectives of the original study were to explore the prevalence of MSIMI in ANOCA and the underlying blood flow mechanisms. Additionally, baseline and post-MS blood samples were collected and analyzed to further investigate the pathophysiology of MSIMI. The present analysis represents a prespecified component of this overall study and was designed to examine whether an association exists between baseline inflammatory status and MS-induced myocardial perfusion defects. Proteomic profiling of plasma samples was additionally conducted to explore potential biological processes underlying MSIMI.

ANOCA was defined as having typical or atypical angina without obstructive coronary artery disease (luminal stenosis ≥50%), confirmed by either coronary angiography (CAG) or coronary computed tomography angiography (CCTA) within the past year (Samuels et al., 2023). Participants were excluded if they had chest pain due to noncardiac circulatory causes, or if they had used antidepressants or antipsychotics within the last month.

All participants underwent three PET/CT scans: resting, MS, and adenosine stress (AS). The resting PET/CT scan could be performed before the stress PET/CT scans, but there was a minimum 30-minute interval between the two. Participants were hospitalized, and sociodemographic and psychosocial data were collected during hospitalization. Standardized MS and AS tests were conducted on separate mornings within a week, in random order. Before these tests, participants were instructed to discontinue beta-blockers and calcium channel blockers for 3 to 5 half-lives and to abstain from tobacco and coffee for at least 12 hours. Participants with contraindications to adenosine, those unable to tolerate it, or those with concerns about potential side effects were exempted from the AS test.

This study was approved by the Medical Research Ethics Committee of Guangdong Provincial People’s Hospital (GDREC 2019298H[R3]) and strictly adhered to the ethical principles of the Declaration of Helsinki. All participants provided written informed consent.

### Mental stress testing

After a 15-minute rest in a quiet, dimly lit room, participants underwent MS testing using a virtual reality (VR) device, which presented three tasks in a fixed sequence: the modified Stroop test, public speaking, and a mental arithmetic test. The entire stress period lasted 12 minutes, and ^13^N-ammonia was injected 5–8 minutes after the start of MS testing. During the PET scan, electrocardiogram and heart rate were continuously monitored, and blood pressure was recorded every minute (see Supplementary Material for the detailed protocol of MS testing).

### Adenosine stress testing

The AS testing protocol followed the recommendations of the American Society of Nuclear Cardiology, with adenosine administered at a rate of 140 μg/kg/min (100–120 μg/kg/min for those at risk of complications) for 6 minutes. ^13^N-ammonia was injected at the beginning of the fourth minute after adenosine infusion began.

### Myocardial perfusion imaging and quantitative myocardial blood flow

The severity of myocardial ischemia (perfusion defects) was evaluated for each state using a 17-segment AHA-defined left ventricular model and a semi-quantitative scoring system. Scores ranged from 0 (normal) to 4 (no perfusion). The summed stress score (SSS), summed resting score (SRS), and summed difference score (SDS, SDS = SSS - SRS) were calculated. Total perfusion defect (TPD) is an automated image-based quantitative method for assessing the percentage of the entire myocardium with perfusion defects. It uses automated tools to compare patient images with a reference database of healthy individuals, quantifying the extent of myocardial perfusion defects. Although TPD is more objective, its accuracy may be affected by large chest wall motion (e.g. during MS), whereas SDS, which involves manual delineation of segmental regions by radiologists, can be more precise. To reduce the subjectivity of SDS, PET images were analyzed by two experienced nuclear cardiologists blinded to the conditions. The images were checked for errors, such as artifacts or low count density, before interpretation. Based on previous studies, MSIMI was defined as an SDS_MS_ ≥ 3 (Vaccarino et al., 2018).

Myocardial blood flow (MBF) was calculated using Cedars-Sinai software and a two-compartment model based on the time–activity curve of left ventricular input and myocardial uptake obtained within the first 120 seconds after ^13^N-ammonia injection. Coronary flow reserve (CFR) was determined as the ratio of MBF_AS_ to MBF_rest_. Coronary microvascular disease was defined as CFR < 2.0. Summed motion score (SMS), which indicates the extent of abnormal wall motion, and cardiac function measurements were also automatically calculated.

### Inflammatory biomarker measurements

Baseline blood samples were collected between 6 and 7 AM on the second day after admission. All samples were sent to the hospital laboratory for analysis before 8 AM. The inflammatory markers measured in this study included IL-6, CRP, high-sensitivity CRP (hsCRP), a complete blood count (including white blood cells, neutrophils, and lymphocytes), ceruloplasmin, α1-acid glycoprotein, and immune markers (complement component 3 (C3), complement component 4 (C4), and immunoglobulins (IgG, IgA, IgM)). IL-6 was not measured in control subjects. We also assessed cardiac injury markers, including high-sensitivity troponin (hs-TNT) and N-terminal pro-B-type natriuretic peptide (NT-proBNP).

The normal reference ranges used in this study were derived from our hospital laboratory’s validated standards, which are established based on the specific reagents, assay platforms, and quality-controlled local population data used in routine clinical testing. The laboratory-defined normal ranges were as follows: IL-6, 0–7 pg/mL; hsCRP, 0–10 mg/L; CRP, 0–5 mg/L; and C3, 900–1800 mg/L (more details in Supplementary Table S1). Values below the lower limits of detection (IL-6 1.5 pg/mL; hsCRP 0.15 mg/L; CRP 0.3 mg/L) were observed in 19 (15.6%), 5 (4.10%), and 14 (11.5%) participants, respectively; no C3 values were undetectable. Detection limit values were used for imputation. Four participants in the ANOCA group declined the inflammatory biomarker blood draw, resulting in a sample size of 80 for the ANOCA group and 42 for the control group. All test results from the participants were within or very slightly above the normal reference range.

### Liquid chromatography mass spectrometry/mass spectrometry (LC–MS/MS) analysis

To investigate the involvement of biological processes in MSIMI, proteomic analysis was performed on blood samples collected 30 minutes following the end of MS test (Ma et al., 2020, 2023). Given the focus of this study and the complex pathogenesis of MSIMI, we selected five MSIMI-positive (MSIMI+) ANOCA participants with the highest IL-6 levels, five MSIMI-negative (MSIMI-) ANOCA participants with the lowest IL-6 levels, and five healthy controls for comparative proteomic analysis.

The whole blood samples were centrifuged at 1500 g for 10 minutes at 4 °C, after which the plasma was aliquoted and stored at −80 °C for further analysis.

Plasma proteins were captured by incubating zeolite material, pre-washed with deionized water and methanol, with plasma in buffer A. After washing, proteins were reduced, alkylated, and digested with trypsin. Peptides were desalted, quantified, and analyzed by LC–MS/MS using an EASY-nLC 1200 coupled to an Orbitrap Eclipse. Peptides were separated on a C18 column with a solvent B gradient, and high-resolution MS1 scans of intact peptides and MS2 scans of their fragments were acquired for identification. Data were processed with DIA-NN v1.8; proteins with >50% missing values in any group were removed, missing values imputed by KNN (K = 10), and Z-score normalization applied. Differentially expressed proteins (DEPs) were identified using Limma with p < 0.05 and absolute fold-change >2.

### Statistical analysis

Continuous variables are presented as mean ± SD or median (IQR), as appropriate, and categorical variables as counts (percentages). Within-group comparisons of hemodynamic and PET/CT-derived indices across rest, MS, and AS were performed using paired t tests or Wilcoxon signed-rank tests. MBF was corrected for cardiac workload by dividing MBF by the rate–pressure product (RPP) at rest and during MS. Between-group comparisons (MSIMI+ versus MSIMI-) were conducted using Student’s t test or the Mann–Whitney U test for continuous variables and the χ^2^ test or Fisher’s exact test for categorical variables. Associations between baseline inflammatory biomarkers and MS-induced myocardial perfusion abnormalities were evaluated using Spearman correlation analyses. Multivariable logistic regression models were constructed to assess the association between inflammatory biomarkers and the presence of MSIMI, with sequential adjustment for demographic and clinical covariates and resting MBF. Sensitivity analyses modeled IL-6 as a dichotomous variable, a log2-transformed continuous variable, and a rank-transformed variable. Generalized linear models were used to examine associations with perfusion defect severity (SDS or TPD). All tests were two-sided with α = 0.05. Statistical analyses were performed using SAS 9.4.

For proteomic analyses, DEPs were identified using criteria of fold-change (FC) ≥ 1 and *p* < 0.05. Gene ontology (GO) enrichment analysis incorporating fold-change information from all detected proteins was performed to identify biological processes, cellular components, and molecular functions associated with differential protein expression. Kyoto Encyclopedia of Genes and Genomes (KEGG) pathway enrichment analysis was subsequently conducted to characterize altered signaling and metabolic pathways. Protein–protein interaction (PPI) networks were constructed, and density-based clustering (DBSCAN) was applied to identify functionally related protein modules underlying MSIMI. All analyses, including DEP analysis, GO and KEGG enrichment were performed in R (version 4.2.3) using the DEP and clusterProfiler packages, with PPI and DBSCAN analyses conducted via the STRING database (https://string-db.org).

## Results

### Mental stress-induced myocardial perfusion defects in ANOCA patients

Among the 80 participants with ANOCA, 34 (42.5%) were diagnosed with MSIMI, while only 1 (2.4%) participant in the control group had MSIMI. There were no statistically significant differences in demographic and clinical characteristics between ANOCA patients with and without MSIMI (Table 1).

**Table 1.** Demographic and clinical characteristics of participants

	ANOCA			Control	
	MSIMI+ (N = 34)	MSIMI− (N = 46)	*p* value^a^	N = 42	*p* value^b^
Age, years	54.1 ± 9.0	53.4 ± 8.5	0.728	53.4 (8.5)	0.935
Educational level, n (%)			0.133		0.734
Primary school	3 (8.8)	8 (17.4)		7 (16.7)	
Middle school	7 (20.6)	12 (26.1)		6 (14.3)	
Senior middle school	9 (26.5)	12 (26.1)		14 (33.3)	
Graduate and above	15 (44.1)	14 (30.4)		15 (35.7)	
Income per month, n (%)			0.215		0.933
<3000¥	9 (26.5)	18 (39.1)		12 (28.6)	
3000–7000¥	13 (38.2)	17 (37.0)		18 (42.9)	
7000–20000¥	8 (23.5)	7 (15.2)		10 (23.8)	
>20000¥	4 (11.8)	4 (8.7)		2 (4.8)	
Married, n (%)	32 (94.1)	41 (89.1)	0.693	36 (85.7)	0.469
Menstrual history: menopausal, n (%)	22 (64.7)	30 (65.2)	0.962	28 (66.7)	0.792
CCS angina rating, n (%)			0.180		<0.001
I	22 (64.7)	36 (78.3)		0 (0)	
II	12 (35.3)	10 (21.7)		0 (0)	
Angiographic data, n (%)			0.235		0.722
Without visible stenosis	26 (76.5)	40 (87.0)		36 (85.7)	
Maximum stenosis ≤30%	5 (14.7)	4 (8.7)		4 (9.5)	
30% < Maximum stenosis <50%	3 (8.8)	2 (4.3)		2 (4.7)	
Psychological profile					
HADS-depression score	6.0 (3.0, 9.0)	6.5 (3.0, 9.0)	> 0.99	2.5 (1.0–5.0)	<0.001
HADS-anxiety score	7.5 (6.0, 10.0)	7.0 (5.0, 10.0)	0.431	3.0 (1.0–5.0)	<0.001
PSS-14 score	26.5 (20.0, 32.0)	27.0 (21.0, 32.0)	0.682	18.0 (15.0–22.0)	<0.001
Comorbid disorders, n (%)					
Hypertension	9 (26.5)	11 (23.9)	0.794	1 (2.4)	0.002
Diabetes	3 (8.8)	4 (8.7)	> 0.99	1 (2.4)	0.196

*Note:* Continuous variables are represented as mean ± SD or median (IQR). ANOCA, angina with no obstructive coronary artery disease; MSIMI, mental stress-induced myocardial ischemia; CCS, Canadian Cardiovascular Society; HADS, hospital anxiety and depression scales; PSS, perceived stress scale; SD, standard deviation; IQR, interquartile range.

a *p* value for comparisons between participants with and without MSIMI in ANOCA group.

b *p* value for comparisons between ANOCA group and Control group.

The semi-quantitative perfusion defect scores and quantitative TPD scores significantly increased under MS. ANOCA participants with MSIMI (SDS 4.83 [IQR 4.10–5.56], TPD 5.25 [IQR 4.34–6.16]) exhibited a markedly greater increase in perfusion defect compared with those without MSIMI (SDS 0.63 [IQR 0.40–0.85], TPD 1.31 [IQR 0.93–1.70]) (all *p* < 0.001) (Table 2). Both MSIMI+ and MSIMI- participants showed significant increases in MBF under MS compared with baseline. Considering the increased cardiac workload and oxygen demand, corrected MBF, after adjusting for RPP, showed a notable decrease in myocardial blood supply relative to cardiac work during MS testing. MSIMI+ participants had a lower corrected MBF (0.96 ± 0.23 ml/g/mmHg) compared with MSIMI- participants (1.05 ± 0.29 ml/g/mmHg), but this difference was not statistically significant.

**Table 2.** Comparison of PET/CT measurements between ANOCA participants with and without MSIMI

	ANOCA MSIMI+		ANOCA MSIMI−	
	Resting state	MS state	Resting state	MS state
Myocardial perfusion imaging				
Semi-quantitative defect score	0.58 ± 1.34	5.42 ± 2.35	0.42 ± 0.68	1.04 ± 1.05
SDS (△perfusion defect score)	4.83 (4.10, 5.56) ^***^		0.63 (0.40, 0.85) ^***^	
Total perfusion defect (TPD)	0.50 ± 1.16	5.57 ± 2.73	0.15 ± 0.41	1.46 ± 1.40
△TPD	5.25 (4.34, 6.16) ^***^		1.31 (0.93, 1.70) ^***^	
Myocardial MBF quantification				
MBF, ml/g/min	1.03 ± 0.21	1.25 ± 0.39	1.09 ± 0.32	1.29 ± 0.48
CFR	2.41 ± 0.75		2.66 ± 0.81	
Hemodynamic measurements				
Heart rate, beat/min	69 ± 10	91 ± 22	70 ± 12	94 ± 22
Systolic blood pressure, mmHg	115 ± 12	142 ± 23	115 ± 13	140 ± 20
△Rate–pressure product, mmHg/min	5570 (4160, 6980) ^***^		4679 (3904, 5454) ^***^	
Balance between blood supply and oxygen demand				
Corrected MBF, ml/g/mmHg	1.30 ± 0.29	0.96 ± 0.23	1.37 ± 0.34	1.05 ± 0.29
△Corrected MBF, ml/g/mmHg	−0.34 (−0.45, −0.24) ^***^		−0.31 (−0.42, −0.21) ^***^	
Cardiac function assessment				
Wall motion score	0.69 ± 1.43	0.83 ± 1.48	0.20 ± 0.40	0.23 ± 0.47
△EDV, ml	−3.69 (−5.92, 1.47) ^**^		0.09 (−1.25, 1.42)	
△ESV, ml	−2.92 (−4.26, −1.58) ^***^		−1.07 (−1.72, −0.42) ^**^	
△SV, ml	−0.89 (−1.25, 1.42)		1.29 (0.03, 2.25) *	
△LVEF, %	2.50 (1.10, 3.90) ^***^		1.80 (1.01, 2.59) ^***^	

*Note:* Continuous variables are represented as mean ± SD or median (IQR). Changes in mental stress testing are represented as mean (95% CI). *: *p* < 0.05, ^**^: *p* < 0.01, ^***^: *p* < 0.001. Δ indicates the difference between MS and resting state. MBF, myocardial blood flow; CFR, coronary flow reserve; ANOCA, angina with no obstructive coronary artery disease; MSIMI, mental stress-induced myocardial ischemia; MS: mental stress; SDS, summed difference score; EDV, end-diastolic volume; ESV, end-systolic volume; SV, stroke volume; LVEF, left ventricular ejection fraction; SD, standard deviation; CI, confidence interval.

Regarding cardiac function, ANOCA patients under MS experienced a significant decrease in end-systolic volume (ESV) and an increase in ejection fraction (EF), indicating enhanced myocardial contractility. However, the stroke volume (SV) in the MSIMI+ group decreased (−0.89 [IQR -2.67–0.89] ml), while the MSIMI- group showed a significant increase (1.29 [IQR 0.03–2.55] ml).

### Elevated baseline noninfectious inflammation in MSIMI+ ANOCA patients

Cardiac injury markers, including hs-TNT and NT-proBNP, were not elevated in MSIMI+ patients and were not associated with stress-induced myocardial perfusion defects. In addition, no significant differences were observed between MSIMI+ and MSIMI- groups in white blood cell counts, neutrophils, lymphocytes, ceruloplasmin, alpha-1-acid glycoprotein (AAG), IgM, and IgG (Table 3), suggesting no evidence of acute or chronic infectious activation.

**Table 3.** Comparison of inflammatory markers in ANOCA and Control groups

	ANOCA			Control	
	MSIMI+ (N = 34)	MSIMI− (N = 46)	*p* value^a^	N = 42	*p* value^b^
IL-6, pg/mL	3.20 (1.50, 4.90)	1.80 (1.50, 3.30)	**0.024**	–	–
CRP, mg/L	1.25 (0.63, 2.39)	0.81 (0.32, 1.62)	**0.036**	0.65 (0.36, 1.11)	**0.041**
hsCRP, mg/L	1.25 (0.63, 2.04)	0.74 (0.30, 1.32)	0.053	0.49 (0.29, 0.92)	**0.019**
C3, mg/L	1114 (1049, 1226)	1054 (921, 1163)	**0.041**	1073 (1004, 1210)	0.651
C4, mg/L	250 (210, 321)	261 (207, 333)	0.782	223 (203, 278)	0.088
IgM, g/L	1.05 ± 0.52	0.99 ± 0.45	0.627	1.12 ± 0.56	0.293
IgA, g/L	2.30 ± 1.07	2.19 ± 0.77	0.626	2.30 ± 1.50	0.777
IgG, g/L	12.60 ± 1.91	12.70 ± 2.11	0.827	12.60 ± 2.26	0.896
CER, mg/L	317 ± 54	315 ± 55	0.881	294 ± 54	**0.035**
AAG, g/L	0.52 ± 0.15	0.50 ± 0.13	0.459	0.50 ± 0.12	0.607
WBC, 10^9^/L	6.16 ± 1.61	5.91 ± 1.47	0.474	5.51 ± 1.03	0.059
NEU%	55.5 ± 6.9	55.0 ± 8.2	0.756	53.0 ± 6.9	0.124
LYMPH%	33.5 ± 6.3	34.7 ± 7.1	0.448	35.9 ± 6.6	0.165
hsTNT, pg/mL	5.0 (5.0, 6.1)	5.0 (5.0, 6.2)	0.463	5.0 (5.0, 5.9)	0.654
NT-proBNP, pg/mL	38.3 (28.0, 73.8)	44.9 (27.7, 72.3)	0.759	42.2(23.3, 60.5)	0.166

*Note:* Continuous variables are represented as mean ± SD or median (IQR). *p* value marked in bold indicates statistically significant. ANOCA, angina with no obstructive coronary artery disease; MSIMI, mental stress-induced myocardial ischemia; IL-6, interleukin-6; CRP, C-reactive protein; hsCRP, high-sensitivity CRP; C3, complement component 3; C4, complement component 4; IgM, immunoglobulin M; IgA, immunoglobulin A; IgG, immunoglobulin G; CER, ceruloplasmin; AAG, alpha-1-acid glycoprotein; WBC, white blood cell; NEU, neutrophil percentage; hsTNT, high-sensitivity troponin T; NT-proBNP, N-terminal pro-B-type natriuretic peptide; SD, standard deviation; IQR, interquartile range.

a *p* value for comparisons between participants with and without MSIMI in ANOCA group.

b *p* value for comparisons between ANOCA group and control group.

In contrast, several inflammation-related biomarkers were significantly higher at baseline in patients with MSIMI (Table 3). Resting IL-6 levels were elevated in MSIMI+ patients compared with MSIMI- patients (3.20 [IQR 1.50–4.90] versus 1.80 [IQR 1.50–3.30] pg/mL, *p* = 0.024). Similarly, CRP and hsCRP levels were higher in the MSIMI+ group (CRP: 1.25 [IQR 0.63–2.39] mg/L; hsCRP: 1.12 [IQR 0.63–2.04] mg/L) than in the MSIMI- group (CRP: 0.81 [IQR 0.32–1.62] mg/L, *p* = 0.036; hsCRP: 0.74 [IQR 0.30–1.32] mg/L, *p* = 0.053). Complement C3 levels were also significantly increased in MSIMI+ patients (1114 [IQR 1049–1226] mg/L) compared with MSIMI- patients (1054 [IQR 921–1163] mg/L, *p* = 0.041).

### Significant correlation between MS-induced myocardial perfusion defect and baseline inflammatory markers

**Figure 1.**
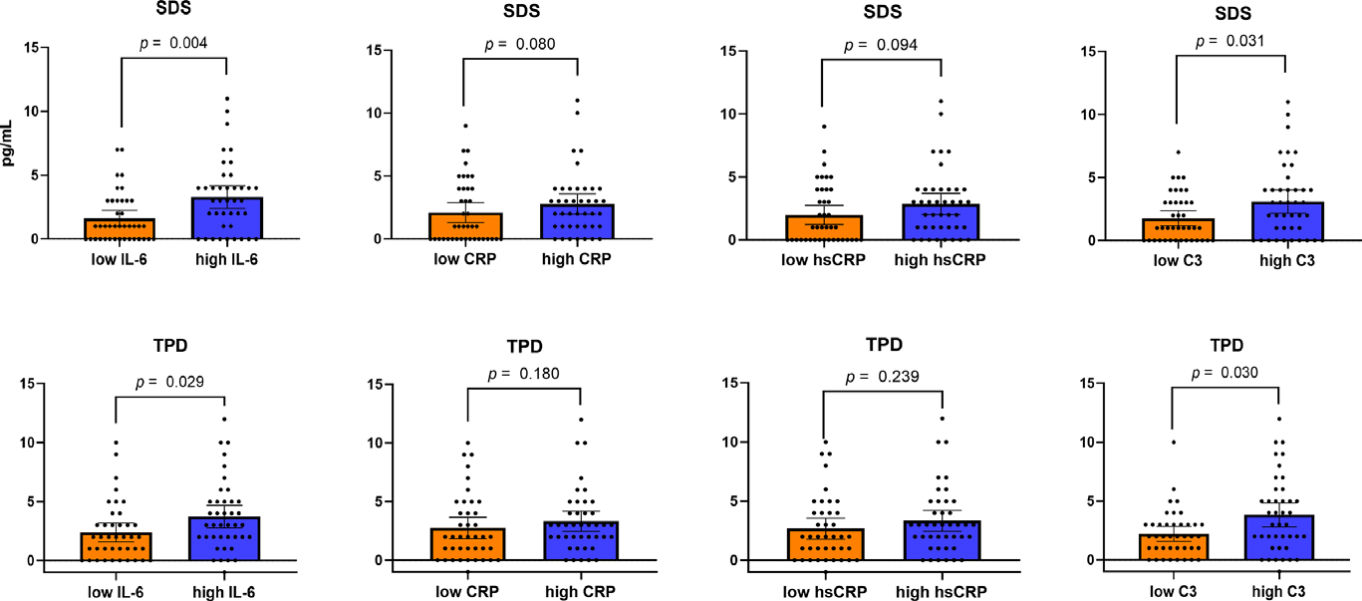
Comparison of myocardial perfusion defects according to dichotomized inflammation markers. Myocardial perfusion defect was assessed using summed difference score (SDS) and total perfusion defect (TPD) across groups defined by IL-6, CRP, hsCRP, and C3 levels. *Note:* IL-6, ‘interleukin-6’; CRP, ‘C-reactive protein’; hsCRP, ‘high-sensitivity CRP’; C3, ‘complement component 3’; SDS, ‘summed difference score’; TPD, ‘total perfusion defect’.

When participants were stratified according to dichotomized baseline inflammatory marker levels, those with higher IL-6 and C3 levels exhibited more severe MS-induced myocardial perfusion defects, whereas CRP and hsCRP were not significantly associated with perfusion defect severity (Figure 1).

Spearman correlation analyses further demonstrated that baseline levels of IL-6, hsCRP, CRP, and C3 were not associated with resting semi-quantitative perfusion scores, but were significantly correlated with perfusion defect scores during MS and with stress-induced changes in perfusion scores (Supplementary Table S2).

In multivariable logistic regression analyses based on dichotomized inflammatory markers (Supplementary Table S3), IL-6 emerged as the only inflammatory biomarker consistently associated with MSIMI among the four inflammation-related indicators examined. This association remained robust across the unadjusted model (OR 3.65, 95% CI [1.47–9.07], *p* = 0.005), the model adjusted for age, body mass index, hypertension, and diabetes history, and the fully adjusted model further accounting for depressive symptoms and resting MBF (adjusted OR 3.61, 95% CI [1.34–9.72], *p* = 0.011). Consistently, in generalized linear models with SDS or TPD as dependent variables (Supplementary Table S3), higher baseline IL-6 levels remained significantly associated with greater severity of MS-induced myocardial perfusion abnormalities.

To address the non-normal distribution of IL-6, additional multivariable analyses were performed with IL-6 modeled as a dichotomous variable, a log_2_-transformed continuous variable, and a rank-transformed variable (Table 4). Both dichotomized and log_2_-transformed IL-6 demonstrated stable and consistent associations with MSIMI across unadjusted and fully adjusted models. Rank-transformed IL-6 showed a similar direction of association and reached either statistical significance or borderline significance, reflecting a consistent trend across modeling approaches.

**Table 4.** Multivariable analysis of the association between myocardial perfusion defect and IL-6 using generalized linear models

	Model 1 (unadjusted)		Model 2		Model 3	
	OR/β (95% CI)	*p* value	OR/β (95% CI)	*p* value	OR/β (95% CI)	*p* value
Predict MSIMI						
IL-6 (high vs low)	3.65 (1.47, 9.07)	**0.005**	3.37 (1.29, 8.84)	**0.014**	3.61 (1.34, 9.72)	**0.011**
IL-6 (log_2_-transformed)	1.65 (1.12, 2.42)	**0.012**	1.69 (1.15, 2.49)	**0.007**	1.82 (1.19, 2.78)	**0.006**
IL-6 (rank-transformed)	1.59 (1.00, 2.54)	0.051	1.56 (0.97, 2.52)	0.068	1.63 (0.98, 2.70)	0.059
Predict SDS						
IL-6 (high vs low)	0.70 (0.25, 1.14)	**0.002**	0.60 (0.15, 1.04)	**0.008**	0.60 (0.15, 1.06)	**0.010**
IL-6 (log_2_-transformed)	0.29 (0.12, 0.46)	**<0.001**	0.30 (0.13, 0.46)	**<0.001**	0.31 (0.14, 0.48)	**<0.001**
IL-6 (rank-transformed)	0.27 (0.04, 0.50)	**0.022**	0.24 (0.02, 0.47)	**0.034**	0.25 (0.01, 0.48)	**0.039**
Predict TPD change						
IL-6 (high vs low)	0.62 (0.19, 1.05)	**0.005**	0.59 (0.15, 1.02)	**0.008**	0.61 (0.17, 1.05)	**0.007**
IL-6 (log_2_-transformed)	0.25 (0.08, 0.41)	**0.003**	0.26 (0.10, 0.42)	**0.001**	0.28 (0.11, 0.44)	**0.001**
IL-6 (rank-transformed)	0.21 (−0.01, 0.43)	0.061	0.21 (−0.01, 0.43)	0.057	0.22 (−0.01, 0.45)	0.054

*Note:* Model 2: Multivariable adjusted effects are adjusted for age, body mass index, hypertension, and diabetes history; Model 3: Multivariable adjusted effects are adjusted for age, body mass index, hypertension, diabetes history, depression, and resting MBF. *p* value marked in bold indicates statistically significant. OR, odds ratio; CI, confidence interval; IL-6, interleukin-6; MSIMI, mental stress-induced myocardial ischemia; SDS, summed difference score; TPD, total perfusion defect; MBF, myocardial blood flow.

### Proteomics insights into possible mechanisms between baseline inflammation and MSIMI

**Figure 2.**
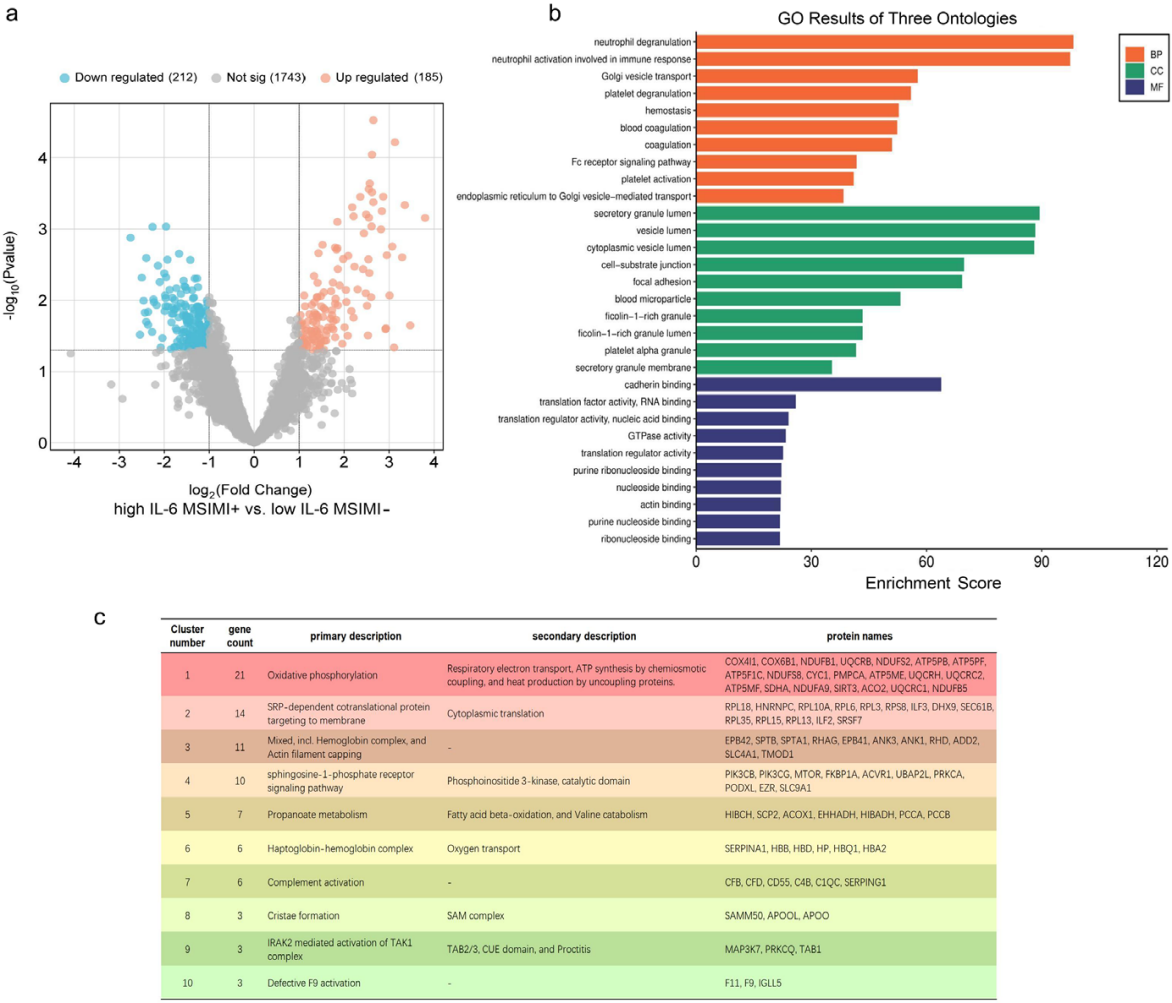
Proteomic analysis of differential protein expression underlying the association between elevated IL-6 levels and MSIMI. (a) Volcano plot showing DEPs between high IL-6 MSIMI+ and low IL-6 MSIMI- patients. (b) GO enrichment analysis of DEPs across three ontologies: biological process, cellular component, and molecular function. (c) DBSCAN-based clustering of the PPI network. GO enrichment analyses and PPI network analysis show enrichment of biological processes related to innate immune–hemostatic pathways and mitochondrial bioenergetics. *Note:* IL-6, ‘interleukin-6’; MSIMI, ‘mental stress-induced myocardial ischemia’; DEP, ‘differentially expressed protein’; GO, ‘gene ontology’; PPI, ‘protein–protein interaction’; DBSCAN, ‘density-based spatial clustering of applications with noise’.

Proteomic analysis identified a total of 397 DEPs between the top five high IL-6 MSIMI+ and the top five low IL-6 MSIMI- ANOCA participants, including 185 upregulated and 212 downregulated proteins (Figure 2a).

Across the three GO ontologies (Figure 2b), the enriched terms converged on two major biological pathways. First, DEPs were predominantly enriched in innate immune–hemostatic pathways, characterized by coordinated activation of neutrophil- and platelet-related processes, including degranulation, coagulation, and Fc receptor–mediated signaling, indicating a coupled immune–thrombotic response. These processes were supported by cellular component enrichment in secretory granules, blood microparticles, and platelet alpha granules, along with molecular function terms related to cytoskeletal organization and cell adhesion. Second, DEPs were enriched in pathways related to mitochondrial bioenergetics, with involvement of oxidative phosphorylation and ATP metabolic processes, and prominent localization to mitochondrial protein complexes and ATP synthase, indicating altered mitochondrial energy metabolism.

Consistent with these findings, KEGG pathway analysis (Supplementary Figure S1A) demonstrated significant enrichment of pathways associated with mitochondrial respiration and cardiac energetics, including pathways annotated as neurodegeneration across multiple diseases. In addition, pathways involved in the complement and coagulation cascades, as well as platelet activation, were significantly enriched, supporting alterations in innate immune–hemostatic signaling. PPI network analysis followed by DBSCAN clustering (Figure 2c, Supplementary Figure S1B) identified a dominant cluster related to oxidative phosphorylation and respiratory electron transport (Cluster 1), along with additional clusters associated with membrane attack complex formation, cytoskeletal regulation, carbon dioxide transport, and ribosomal proteins, corroborating the functional insights from GO and KEGG analyses.

Together, these integrated analyses highlight coordinated alterations in innate immune–hemostatic pathways and mitochondrial bioenergetics in high IL-6 MSIMI+ patients.

## Discussion

Based on a cohort undergoing a standardized MS testing protocol with PET/CT myocardial perfusion imaging, this study establishes an independent and graded association between baseline IL-6 levels and both the risk and severity of MSIMI in women with ANOCA. Small-sample proteomic analyses further suggest downstream involvement of immune–hemostatic activation and altered mitochondrial bioenergetics. Together, these findings implicate IL-6-associated inflammation as a key pathophysiological pathway underlying susceptibility to ischemia during MS and suggest that strategies aimed at reducing baseline inflammatory burden may represent a potential therapeutic avenue for MSIMI.

Chronic, low-grade, noninfectious inflammation has been firmly established as a contributor to increased cardiovascular risk (Manning, Shroff, Jacobs, & Duprez, 2025). IL-6 is critically involved in the initiation and amplification of inflammatory pathways, promoting vascular and systemic inflammation, endothelial dysfunction, and myocardial remodeling in conditions such as atherosclerosis, hypertension, and heart failure (Mehta, deGoma, & Shapiro, 2024). Prior evidence further indicates that IL-6 independently predicts cardiovascular mortality beyond traditional cardiovascular risk factors (Ofstad et al., 2013). In parallel, both acute and chronic psychological stress – including loneliness and poorer psychological status (Hackett et al., 2012; X. Liu et al., 2022) – have been shown to elevate systemic inflammatory activity, particularly IL-6, thereby providing a plausible link between stress exposure and inflammation-related cardiovascular vulnerability.

MSIMI represents an important yet underrecognized phenomenon. It occurs more frequently in individuals with psychosocial vulnerability, including women, those living alone, and patients with depression, anxiety, post-traumatic stress disorder, or cognitive impairment, distinguishing it from traditional cardiovascular risk factors (Raggi, Quyyumi, Henein, & Vaccarino, 2025; Vaccarino & Bremner, 2017). Moreover, the presence of MSIMI confers an approximately twofold higher risk of future cardiovascular events (Wei et al., 2014).

In the present study, using PET/CT imaging combined with a standardized VR-based MS test, we found that women with ANOCA who developed MSIMI – or who exhibited greater MS-induced myocardial perfusion defects – had significantly higher resting levels of IL-6. This effectively links IL-6 and MSIMI, both of which have cardiovascular predictive value beyond traditional risk factors. These findings are consistent with a previous study of 83 patients with stable CAD, in which 30 participants developed MSIMI, and each 1 mg/L increase in CRP was associated with a 20% higher risk of MSIMI (Shah et al., 2006). Notably, in our study, IL-6 appears to have better predictive value than CRP.

It is worth noting, however, that Hammadah et al. reported in CAD patients that MS induced acute increases in IL-6 and CRP, but no significant differences were observed at baseline or during the 90-minute stress period (Hammadah et al., 2018). Nevertheless, in the validation cohort of that research, they also observed numerically higher median IL-6 and hsCRP levels in MSIMI+ compared to MSIMI- patients, although these differences did not reach statistical significance.

Although MSIMI is diagnosed based on myocardial perfusion defects provoked by acute MS, accumulating evidence indicates that individuals who develop MSIMI already exhibit adverse pathological and physiological alterations at rest. For example, these alterations include elevated EAS index reflecting impaired diastolic function (Ersbøll et al., 2014), impaired CFR suggesting microvascular dysfunction (Yin et al., 2024), and higher levels of depression and anxiety. During acute MS, rapid changes in endothelial function, coronary microvascular spasm, and transient microthrombotic activity may occur (Lima et al., 2019; van der Meer & Maas, 2021). Superimposed on preexisting baseline abnormalities – such as metabolic and neuroendocrine dysregulation and chronic microvascular dysfunction – these stress-induced responses collectively contribute to the development of myocardial perfusion defects.

Elevated IL-6 levels associated with MSIMI may arise through several interconnected mechanisms:

First, elevated IL-6 may directly contribute to MSIMI by promoting coronary microvascular dysfunction and impairing endothelial-dependent vasodilation, which are central pathophysiological features in patients with ANOCA (Del Buono et al., 2021; Ullah et al., 2025). In addition, IL-6 responsiveness may become sensitized through repeated exposure to psychosocial stress in daily life, resulting in chronic endothelial injury and a reduced threshold for maladaptive vascular responses to acute stress, thereby increasing susceptibility to ischemic events (Zannas et al., 2020). Consistent with this concept, prior studies have shown that in patients with stress-induced reversible myocardial ischemia, higher IL-6 levels during peak stress and recovery are associated with both the extent of ischemic myocardial segments and the severity of left ventricular dysfunction (Ikonomidis et al., 2005).

Furthermore, our proteomic analyses demonstrated significant enrichment of innate immune and hemostatic pathways in MSIMI-positive ANOCA patients with high IL-6 levels, supporting a link between inflammation and thrombogenic activation. Previous studies have reported that women with MSIMI exhibit heightened platelet aggregation responses to stress-related mediators at rest compared with men (Samad et al., 2014), suggesting a sex-specific prothrombotic vulnerability. Importantly, IL-6 signaling has been shown to independently accelerate coagulation (Mutlu et al., 2007), providing a plausible mechanistic bridge between systemic inflammation, platelet activation, and stress-induced ischemia.

Finally, alterations in mitochondrial bioenergetics may represent an additional mechanistic pathway linking elevated IL-6 to MSIMI. Stress-induced IL-6 has been reported to be produced by brown adipocytes in a 3-adrenergic-receptor-dependent fashion (Qing et al., 2020). Inflammatory signaling can impair mitochondrial function and energy metabolism, limiting myocardial metabolic flexibility under stress (Borger et al., 2024). Concurrently, mitochondrial dysfunction and excessive oxidative phosphorylation can increase reactive oxygen species production, amplify inflammation, and further disrupt energy metabolism, leading to endothelial dysfunction (Guo, Sun, Chen, & Zhang, 2013). Together, these effects may heighten myocardial susceptibility to stress-induced ischemia, predisposing vulnerable individuals to transient ischemic events during acute mental challenges (Guo et al., 2013; Madamanchi & Runge, 2007; Xu, Jin, & Weng, 2022).

These findings have several important clinical and mechanistic implications. They support baseline IL-6 as a meaningful biomarker for identifying women with ANOCA who are at heightened risk of MSIMI, extending cardiovascular risk stratification beyond traditional ischemic and inflammatory markers. The observed associations between IL-6, immune–hemostatic activation, and altered mitochondrial bioenergetics suggest that MSIMI reflects a systemic vulnerability characterized by inflammation-driven microvascular, thrombotic, and metabolic dysregulation rather than an isolated stress-provoked phenomenon. Finally, the graded relationship between IL-6 levels and MSIMI severity underscores the potential importance of baseline inflammatory burden in lowering the ischemic threshold during MS. Therapeutic strategies targeting chronic low-grade inflammation – alongside psychosocial stress reduction – may represent a novel approach to preventing stress-induced ischemia and improving cardiovascular outcomes in women with ANOCA.

Although this is the first study to report an association resting inflammatory and MSIMI in women patients with ANOCA, there are several limitations that warrant discussion. Firstly, this is a single-center clinical study involving only women, with a relatively small sample size, which may limit the generalizability of the findings. Nevertheless, given prior evidence that women are more susceptible to MSIMI, focusing on this population is justified. Secondly, the cross-sectional design of this study limits the ability to infer causality between resting IL-6 levels and MSIMI. Elevated IL-6 may contribute to the development of MSIMI, while stress-induced ischemic episodes may, in turn, amplify systemic inflammatory responses. Thirdly, inflammatory levels were measured only at rest, which may have limited the characterization of the dynamic inflammatory response associated with MSIMI. Furthermore, the proteomic analysis was performed on a relatively small subset of participants and limited to peripheral blood samples, which may not fully represent myocardial or vascular mechanisms underlying MSIMI.

## Conclusions

This study demonstrates an independent and graded association between resting IL-6 levels and MS-induced myocardial perfusion defect in women with ANOCA. IL-6 levels may serve as a useful biomarker for identifying individuals at increased risk of MSIMI. Proteomic evidence of immune–hemostatic activation and altered mitochondrial bioenergetics supports a systemic, inflammation-driven mechanism and highlights chronic inflammation as a potential therapeutic target.

## Supporting information

10.1017/S0033291726103638.sm001 Yin et al. supplementary material Yin et al. supplementary material

## Abbreviations

AAG: alpha-1-acid glycoprotein
ANOCA: angina with no obstructive coronary artery disease
AS: adenosine stress
C3: complement component 3
C4: complement component 4
CAD: coronary artery disease
CAG: coronary angiography
CCTA: coronary computed tomography angiography
CFR: coronary flow reserve
CRP: C-reactive protein
DEPs: differentially expressed proteins
EF: ejection fraction
ESV: end-systolic volume
FC: fold-change
GO: gene ontologyhs
CRP: high-sensitivity CRPhs-TNT high-sensitivity troponin
IQR: interquartile range
KEGG: Kyoto Encyclopedia of Genes and Genomes
IL-6: interleukin-6MBF myocardial blood flow
MS: mental stress
MSIMI: mental stress-induced myocardial ischemia
NT-proBNP: N-terminal pro-B-type natriuretic peptide
PET/CT: positron emission tomography/computed tomography
RPP: rate–pressure product
SD: standard deviation
SDS: summed difference score
SMS: summed motion score
SRS: summed resting score
SSS: summed stress score
SV: stroke volume
TPD: total perfusion defect
VR: virtual reality

## Supplementary material

The supplementary material for this article can be found at http://doi.org/10.1017/S0033291726103638.
