# Supplementary material for: Dose-dependent association between elevated resting IL-6 levels and severity of mental stress-induced myocardial perfusion defects
Source: Psychol Med. 2026 Apr 7;56:e94. doi: 10.1017/S0033291726103638 (PMC13079205; doi:10.1017/S0033291726103638)

**Supplementary Material**

**Supplementary Table 1. Normal reference ranges of measured inflammatory and cardiac injury biomarkers.**

|  | **Reference Range** |
| --- | --- |
| **IL-6** | 0-7 pg/mL |
| **CRP** | 0-5 mg/L |
| **hsCRP** | 0-10 mg/L |
| **C3** | 900-1800 mg/L |
| **C4** | 100-400 mg/L |
| **IgM** | 0.4-2.3 g/L |
| **IgA** | 0.7-4.0 g/L |
| **IgG** | 7-16 g/L |
| **CER** | 220-580 mg/L |
| **AAG** | 0.5-1.2 g/L |
| **WBC** | 3.5-9.5 10^9^/L |
| **NEU%** | 40-75 |
| **LYMPH%** | 20-50 |
| **hsTNT** | 0-14 pg/mL |
| **NT-proBNP** | 0-125 pg/mL |

L-6, Interleukin-6; CRP, C-reactive protein; hsCRP, high-sensitivity CRP; C3, complement component 3; C4, complement component 4; IgM, immunoglobulin M; IgA, immunoglobulin A; IgG, immunoglobulin G; CER, ceruloplasmin; AAG, alpha-1-acid glycoprotein; WBC, write blood cell; NEU, neutrophil percentage; hsTNT, high-sensitivity troponin T; NT-proBNP, N-terminal pro-B-type natriuretic peptide.

**Supplementary Table 2. Spearman correlation between myocardial perfusion defect and inflammatory markers in ANOCA patients.**

|  |  | **SRS** | **SSS** | **SDS** | **TPD**  **resting state** | **TPD**  **MS state** | **TPD change** |
| --- | --- | --- | --- | --- | --- | --- | --- |
| **IL-6** | r | 0.033 | 0.307^**^ | 0.327^**^ | 0.075 | 0.242^*^ | 0.198 |
|  | *p* value | 0.774 | 0.006 | 0.003 | 0.514 | 0.031 | 0.083 |
| **hsCRP** | r | 0.038 | 0.288^**^ | 0.270^*^ | 0.009 | 0.168 | 0.195 |
|  | *p* value | 0.735 | 0.009 | 0.015 | 0.935 | 0.135 | 0.085 |
| **CRP** | r | 0.079 | 0.316^**^ | 0.298^**^ | 0.072 | 0.208 | 0.227^*^ |
|  | *p* value | 0.483 | 0.004 | 0.007 | 0.527 | 0.062 | 0.044 |
| **C3** | r | -0.001 | 0.314^**^ | 0.356^**^ | 0.078 | 0.333^**^ | 0.349^**^ |
|  | *p* value | 0.991 | 0.004 | 0.001 | 0.495 | 0.002 | 0.002 |

Note: ^*^: *p* < 0.05, ^**^: *p* < 0.01, ^***^: *p* < 0.001

ANOCA, angina with no obstructive coronary artery disease; SRS, summed resting score; SSS, summed stress score; SDS, summed difference score; TPD, total perfusion defect; MS, mental stress; IL-6, Interleukin-6; hsCRP, high-sensitivity CRP; CRP, C-reactive protein; C3, complement component 3.

**Supplementary Table 3. Multivariable analysis of the association between myocardial perfusion defect and inflammatory markers using generalized linear models.**

|  | **Model 1 (unadjusted)** | | **Model 2** | | | | **Model 3** | | | | |  |
| --- | --- | --- | --- | --- | --- | --- | --- | --- | --- | --- | --- | --- |
|  | **OR / β (95% CI)** | ***p* value** | **OR / β (95% CI)** | | ***p* value** | | **OR / β (95% CI)** | | ***p* value** | | |  |
| **Predict MSIMI** | | | | | | | | | | | |  |
| **IL-6 (high vs low)** | 3.65 (1.47, 9.07) | **0.005** | 3.37 (1.29, 8.84) | | **0.014** | | 3.61 (1.34, 9.72) | | **0.011** | |  |  |
| **CRP (high vs low)** | 1.92 (0.79, 4.66) | 0.151 | 1.61 (0.63, 4.11) | | 0.317 | | 1.76 (0.68, 4.54) | | 0.245 | |  |  |
| **hsCRP (high vs low)** | 1.86 (0.77, 4.47) | 0.167 | 1.56 (0.62, 3.95) | | 0.345 | | 1.66 (0.65, 4.22) | | 0.291 | |  |  |
| **C3 (high vs low)** | 1.80 (0.75, 4.31) | 0.188 | 1.80 (0.75, 4.31) | | 0.280 | | 1.72 (0.66, 4.51) | | 0.271 | |  |  |
| **Predict SDS** | | | | | | | | | | | |  |
| **IL-6 (high vs low)** | 0.69 (0.25, 1.14) | **0.002** | 0.60 (0.15, 1.04) | | **0.008** | | 0.60 (0.15, 1.06) | | **0.010** | |  |  |
| **CRP (high vs low)** | 0.49 (0.02, 0.96) | **0.040** | 0.37 (-0.08, 0.83) | | 0.106 | | 0.37 (-0.09, 0.83) | | 0.115 | |  |  |
| **hsCRP (high vs low)** | 0.46 (0.00, 0.92) | **0.048** | 0.34 (-0.11, 0.97) | | 0.134 | | 0.34 (-0.12, 0.79) | | 0.149 | |  |  |
| **C3 (high vs low)** | 0.44 (-0.01, 0.91) | 0.057 | 0.38 (-0.07, 0.83) | | 0.100 | | 0.41 (-0.05, 0.88) | | 0.081 | |  |  |
| **Predict TPD change** | | | | | | | | | | | |  |
| **IL-6 (high vs low)** | 0.62 (0.19, 1.05) | **0.005** | | 0.59 (0.15, 1.02) | | **0.008** | | 0.61 (0.17, 1.05) | | **0.007** | | |
| **CRP (high vs low)** | 0.30 (-0.16,0.76) | 0.196 | | 0.21 (-0.23, 0.66) | | 0.352 | | 0.19 (-0.26, 0.65) | | 0.402 | | |
| **hsCRP (high vs low)** | 0.25 (-0.19, 0.70) | 0.268 | | 0.15 (-0.29, 0.58) | | 0.513 | | 0.12 (-0.32, 0.57) | | 0.584 | | |
| **C3 (high vs low)** | 0.49 (0.052, 0.93) | **0.028** | | 0.44 (0.01, 0.88) | | **0.046** | | 0.46 (0.01, 0.91) | | **0.044** | | |

Model 2: Multivariable adjusted effects are adjusted for age, body mass index, hypertension, and diabetes history; Model 3: Multivariable adjusted effects are adjusted for age, body mass index, hypertension, diabetes history, depression and resting MBF.

Note: *p* value marked in bold indicates statistically significant.

OR, odds ratio; CI, confidence internal; IL-6, Interleukin-6; CRP, C-reactive protein; MSIMI, mental stress-induced myocardial ischemia; SDS, summed difference score; TPD, total perfusion defect.

**Supplementary Figure 1. Proteomic analysis of differential protein expression underlying the association between elevated IL-6 levels and MSIMI.** (A) Chord diagram illustrating enriched KEGG pathways, incorporating fold-change information from all detected proteins. (B) DBSCAN-based clustering of the PPI network. GO and KEGG enrichment analyses and PPI network analysis show enrichment of biological processes related to innate immune–hemostatic pathways and mitochondrial bioenergetics.

Abbreviations: IL-6, interleukin-6; MSIMI, mental stress–induced myocardial ischemia; DEP, differentially expressed protein; GO, gene ontology; KEGG, kyoto encyclopedia of genes and genomes; PPI, protein-protein interaction; DBSCAN, density-based spatial clustering of applications with noise.

**
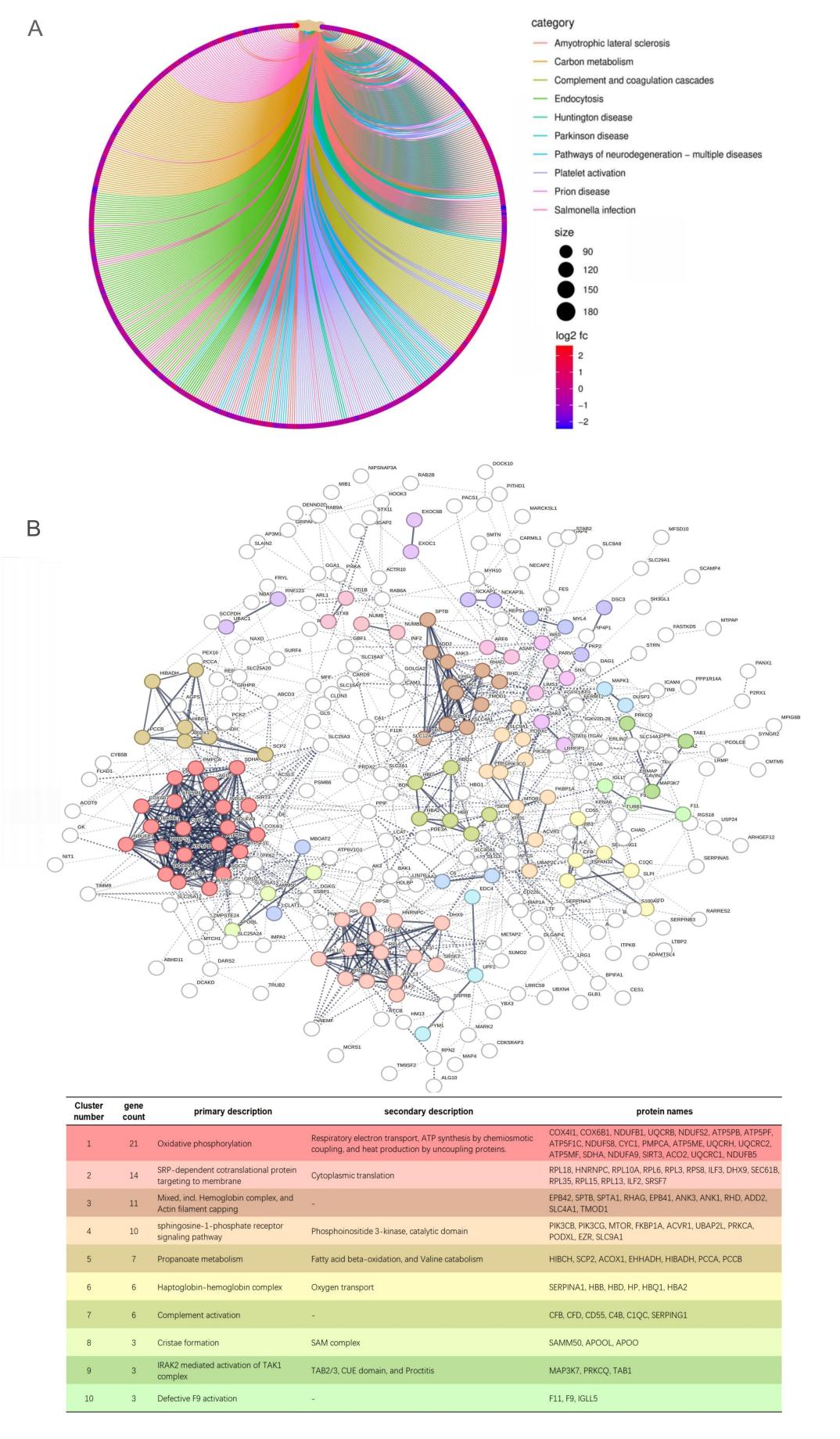
**

**Detailed Protocol of Mental Stress Testing**

The mental stress testing was administered using a virtual reality (VR) device to ensure standardized delivery across participants. On the evening prior to testing, a trained researcher instructed each participant regarding the use of the VR device and provided a brief description of the testing procedures.

On the day of testing, after a 15-minute rest in a quiet, dimly lit room, participants underwent mental stress (MS) testing while wearing a virtual reality device. The device presented three successive tasks in a fixed order: a modified Stroop test, a public speaking task, and a mental arithmetic test.

During the **modified Stroop test**, participants were shown a series of images at gradually decreasing intervals. Each image contained a Chinese character representing a color, while the character itself was displayed in a conflicting color. Participants were required to identify the color of the text and select the correct answer from four options using the VR hand controller. In the **mental arithmetic test**, participants were asked to perform a continuous subtraction task, repeatedly calculating 800 minus 7. For both the Stroop and arithmetic tests, the VR device provided auditory prompts every 20 seconds. Researchers monitored participants’ performance in real time using external equipment and provided verbal encouragement when errors occurred. The **public speaking task** required participants to deliver a speech about a personally difficult, sad, or anger-inducing experience. Participants were given 1 minute to prepare and 3 minutes to speak.

The total duration of the stress period was 12 minutes.^13^N-ammonia was injected 5–8 minutes after the onset of the mental stress testing.

Following the mental stress testing, participants rated task engagement, satisfaction with their performance, and severity of chest pain using a self-administered visual analog scale (VAS) ranging from 1 (lowest) to 10 (highest).


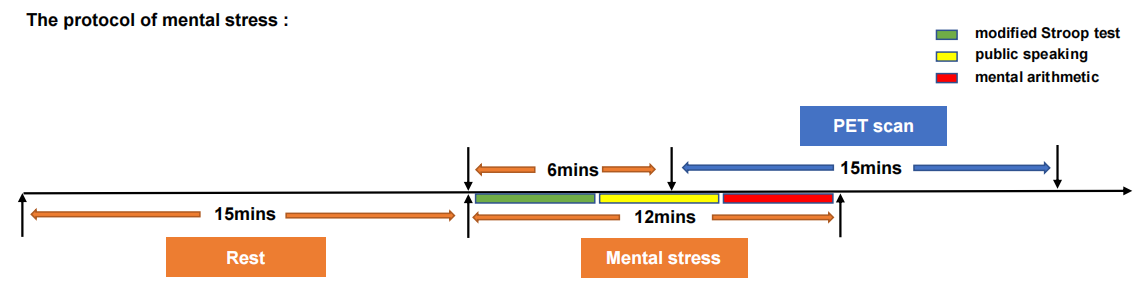


VR device and accompanying mobile applications for mental stress test: (a) MS test preparation; (b) VR-compatible mobile application; (c) VR integrated MS task interface: (left) modified Stroop test, (right) public speaking test. Abbreviation: VR: virtual reality; MS: mental stress.

1.
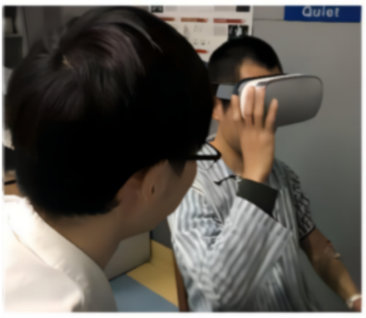

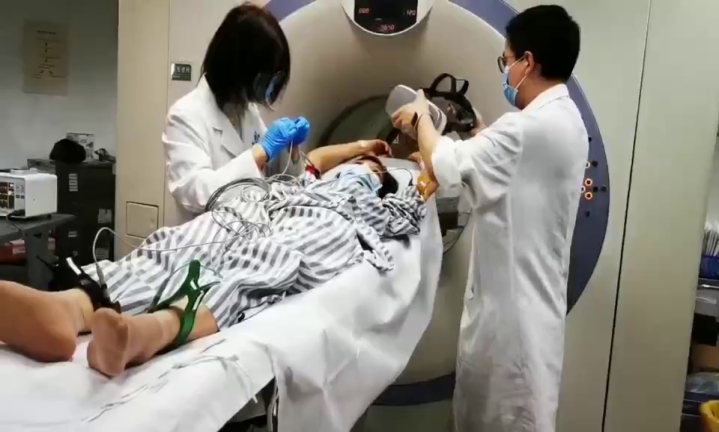

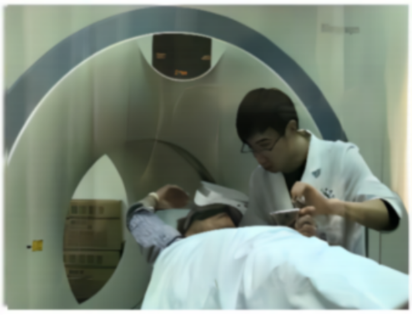

2.
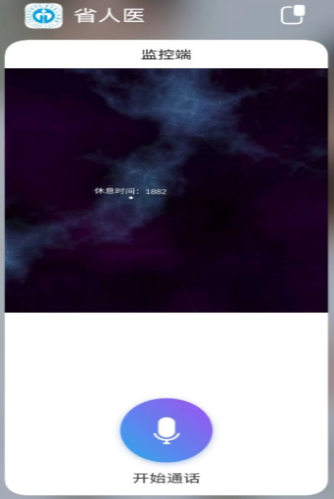
 (c)
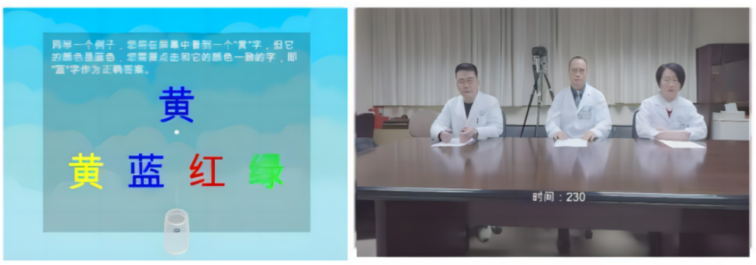

Supplement: Yin et al. supplementary material [file S0033291726103638sup001.docx]
